# Crysalis: an integrated server for computational analysis and design of protein crystallization

**DOI:** 10.1038/srep21383

**Published:** 2016-02-24

**Authors:** Huilin Wang, Liubin Feng, Ziding Zhang, Geoffrey I. Webb, Donghai Lin, Jiangning Song

**Affiliations:** 1 Department of Chemical Biology, College of Chemistry and Chemical Engineering, Xiamen University, Xiamen 361005, China; 2 State Key Laboratory of Agrobiotechnology, College of Biological Sciences, China Agricultural University, Beijing 100193, China; 3 Monash Centre for Data Science, Faculty of Information Technology, Monash University, Melbourne, VIC 3800, Australia; 4 Department of Microbiology, School of Biomedical Sciences, Monash University, Melbourne, VIC 3800, Australia; 5 National Engineering Laboratory for Industrial Enzymes, Tianjin Institute of Industrial Biotechnology, Chinese Academy of Sciences, Tianjin 300308, China

## Abstract

The failure of multi-step experimental procedures to yield diffraction-quality crystals is a major bottleneck in protein structure determination. Accordingly, several bioinformatics methods have been successfully developed and employed to select crystallizable proteins. Unfortunately, the majority of existing *in silico* methods only allow the prediction of crystallization propensity, seldom enabling computational design of protein mutants that can be targeted for enhancing protein crystallizability. Here, we present Crysalis, an integrated crystallization analysis tool that builds on support-vector regression (SVR) models to facilitate computational protein crystallization prediction, analysis, and design. More specifically, the functionality of this new tool includes: (1) rapid selection of target crystallizable proteins at the proteome level, (2) identification of site non-optimality for protein crystallization and systematic analysis of all potential single-point mutations that might enhance protein crystallization propensity, and (3) annotation of target protein based on predicted structural properties. We applied the design mode of Crysalis to identify site non-optimality for protein crystallization on a proteome-scale, focusing on proteins currently classified as non-crystallizable. Our results revealed that site non-optimality is based on biases related to residues, predicted structures, physicochemical properties, and sequence loci, which provides in-depth understanding of the features influencing protein crystallization. Crysalis is freely available at http://nmrcen.xmu.edu.cn/crysalis/.

Knowledge of protein 3D structures has significantly accelerated scientific discovery in many research areas, including protein biological function, drug screening and design, and human health and disease^1^. However, experimental efforts involving protein crystallization frequently fail during the multi-step procedures required to yield diffraction-quality crystals, achieving an overall success rate of only 2–10%^2^. In our recent work, we grouped five major experimental procedures, including sequence cloning failure (CLF), protein material production failure (MF), purification failure (PF), crystallization failure (CF), and ultimately structure determination (CRYs)^3^, and addressed the task of predicting protein crystallization propensity using machine-learning approaches to score each protein on its propensity to successfully pass each of the five steps in the crystallization pipeline. To surmount obstacles that arise from each procedure and accelerate the process, it is necessary to develop a systematic tool that can enable rapid prioritization and selection of appropriate target proteins from a large pool of candidates and allow the rational design of those targets with predicted lower crystallization propensity in order to enhance their crystallizability.

A number of computational approaches have been developed to predict protein crystallization propensity for the purpose of target prioritization and selection using machine learning or statistical algorithms. The first set of computational methods, including SECRET^4^, CRYSTALP^5^, OBScore^6^, ParCrys^7^, CRYSTALP2^8^, MCSG-Z score^9^, and SCMCRYS^10^, adopted a number of sequence-derived features to train prediction models and achieved reasonable computational efficiency. However, when evaluated on an updated independent test dataset, almost all of these methods perform poorly^3^. To improve prediction performance, a second set of computational methods, including XtalPred^11^, Pxs^12^, SVMCRYS^13^, PPCPred^2^, XANNPred^14^, RFCRYS^15^, CRYSpred^16^, and XtalPred-RF^17^, utilize predicted structure information, such as predicted secondary structure, disordered segments, and solvent accessibility of residues. Nevertheless, most state-of-the-art methods, including PPCPred^2^, XtralPred-RF^17^, and PredPPCrys^3^, have computational inefficiencies that limit their capacity to select crystallizable target proteins at proteome-wide scale.

It remains a significant challenge for structural genomics consortiums to crystalize and solve the 3D structures of proteins that possess important biological functions, but are difficult to crystalize^1^,^18^. It has become common practice for structural biologists to introduce one or more point mutations into the wild-type variant in order to generate protein mutants capable of being crystalized and refined^19^,^20^. This strategy is particularly helpful in overcoming obstacles that impede the crystallization of some target proteins, but often requires a trial-and-error approach, which is labor-intensive and time-consuming. Furthermore, the identification of candidate amino acids or regions that can be targeted for site-directed mutagenesis is non-trivial. As a useful alternative, bioinformatics tools can be used as a powerful method to enable rapid *in silico* screening and computational design of those target proteins that are predicted to have lower crystallization propensity. For example, computational methods that provide predictions concerning loops, disordered regions, and solvent-exposed hydrophobic segments/loops help to uncover a source of microheterogeneity that disfavors intermolecular interactions and impedes formation of well-ordered crystals^20^,^21^. Another computational method developed based on the concept of surface entropy reduction (SER) has significantly contributed to the crystallization and structure determination of 15 previously non-crystallizable proteins^22^,^23^.

Here, we develop and implement a new machine learning-based bioinformatics tool called Crysalis, which not only provides rapid prediction of sequence-based protein crystallization propensity, but also enables users to computationally analyze and design protein mutants to further enhance their likelihood of successful crystallization. Crysalis is an updated version of PredPPCrys^3^. It adopts an integrated approach to predicting the propensity of a protein to pass each of the five steps CLF, MF, PF, CF, CRYs using multifaceted sequence-based features and adopting multi-step feature selection to assemble an optimal feature set for each prediction class. This method constructs two-level support-vector regression (SVR) models to estimate the propensity of five experimental procedures in X-ray crystallography-based protein structure determination. Based on the Crysalis prediction method, we further developed the Crysalis design method to facilitate computational engineering of non-crystallizable proteins and integrate a guide annotation of predicted target protein structure characteristics. To confirm Crysalis functions, we validated the Crysalis design method using both the datasets of currently non-crystallizable proteins and experimental protein mutations. In summary, Crysalis is a powerful tool for computational prioritization and the rational design of crystallizable protein targets.

## Results

### Multi-step feature selection

We extracted a total of 4706 multifaceted sequenced-based features to investigate the most important characteristics that determinate the success of five procedures related to X-ray crystallography-based experiments. These feature sets can be categorized into four broad categories (see Methods), as the features based on amino acid composition (AAc), amino acid index (AAindex), k-spaced amino acid pairs (KSAAP), and grading KSAAP (GKSAAP). As described for PredPPCrys^3^, the AAindex-based features utilized 531 complete indices of 20 amino acids available in the current AAindex1 database^24^, which encode abundant physicochemical properties of the target protein^3^,^24^ and display a high correlation coefficient. The KSAAP- and GKSAAP-based features are high-dimensional and high-correlation methods, and a large proportion of these features are irrelevant for the prediction task. Therefore, we performed first-step minimum-redundancy maximum-relevance (mRMR) feature selection on each AAindex-, KSAAP-, and GKSAAP-based feature set and ultimately selected 100 features for each feature group. Next, we performed the second-step mRMR feature selection to filter out other irrelevant/redundant features in the remaining set^3^. The feature distributions of four groups after two-step mRMR for five-class prediction are shown in Table 1.

After the second-step feature selection using mRMR, we selected the top 100 features considering both maximum relevance and minimum redundancy for each prediction class. Subsequently, we adopted forward-feature selection (FFS)^3^,^25^,^26^ to avoid over-fitting of machine-learning models and selected a subset of optimal features. The FFS process was performed using the benchmark training datasets with 5-fold cross-validation and the performances of the corresponding SVR models were evaluated via area under curve (AUC) scores. Ultimately, a smaller subset of the final optimal features for each experimental procedure was generated after the aforementioned multi-step feature selection (Table 1).

Prediction performance of first-level Crysalis models based on benchmark datasets. As described in methods, Crysalis adopts a two-level approach, where the first level predicts a propensity score for each class and then the second level uses the scores for all classes as inputs to the final predictor for the each class. We evaluated the prediction performance of first-level Crysalis SVR models (Crysalis I) using the final optimal feature sets from the benchmark datasets via the 5-fold cross-validation tests. Finally, Crysalis I achieved AUC scores of 0.732, 0.767, 0.790, 0.737, and 0.773 for the prediction of CLF, MF, PF, CF, and CRYs, respectively. We compared the performance of first-level predictors between Crysalis and our previously developed method, PredPPCrys^3^, by assessing six different measures, including AUC, Matthew’s correlation coefficient (MCC), accuracy (ACC), specificity (SPE), sensitivity (SEN), and precision (PRE). The first-level Crysalis SVR predictor performed better than PredPPCrys in terms of the AUC, ACC, and MCC measurements (Table 2), except for the MF class.

### Feature importance and contribution

As described, a small subset of the final optimal features was generated following multi-step feature selection, constituting 25, 78, 95, 68, and 65 features in the final selected feature set for the classes of CLF, MF, PF, CF, and CRYs, respectively (Table 1). We analyzed the contributing and determinant effects of the optimal selected features on the performance of first-level Crysalis SVR predictors. It is evident that the major contributing features are GKSAAP-, KSAAP-, and AAindex-based feature sets based on the results of the feature distribution in the final optimal feature set (Table 1).

Our method distinguishes itself from previous methods in its use of features based on both KSAAP and GKSAAP. Previous studies clarified that the frequency of amino acid pairs is important for protein crystallization prediction^5^,^10^. Our experimental results indicate that the KSAAP-based feature group is another dominating feature set in the final optimal features of all five-class predictions, which agrees with those studies. KSAAP composition was first proposed by Chen *et al.*^27^. Given its significant improvement in sequence-based model performance, the KSAAP encoding scheme has been widely used in a number of bioinformatics studies, such as predictions of flexible/rigid regions^27^, O-glycosylation sites^28^, ubiquitination sites^29^,^30^, protein phosphorylation sites^31^, etc. Nevertheless, for a sequence consisting of 200 amino acids, the average value of all amino acid pairs for KSAAP encoding is only ~0.5. As a result, most amino acid pairs would have little or no statistical significance. Additionally, many amino acid pairs exhibit similar physicochemical properties (e.g. the amino acid pairs KD and RD have similar properties in terms of ion pairs). Accordingly, it is difficult for the KSAAP feature to fully describe and capture all useful information for those similar amino acid pairs. To address this issue, we separated all 20 amino acids into three classes based on their physicochemical properties. Specifically, six significant physicochemical characteristics were graded and applied based on accessible surface area, side-chain orientation, charge, hydrogen-bond donor, hydrophobicity, and van der Waals potential. Importantly, the GKSAAP feature is a new, informative feature developed in this study and plays a role in the predictions of MF and CRYs, resulting in a larger number of final selected features for these two classes. Feature selection results suggest that GKSAAP is a useful feature for the prediction of protein crystallizability (the classes of MF, PF, and CRYs), possibly due to its advantage when capturing useful information involving protein characteristics. Compared with the KSAAP encoding scheme, the novel GKSAAP scheme takes into consideration grading amino acid pairs, not only incorporating pairs that possess similar properties, but also extracting their multifaceted physicochemical properties, rather than those of the individual 20 amino acids.

Previous methods for protein crystallization prediction, such as PPCPred^2^, CRYSPred^16^, and PredPPCrys^3^, used the AAindex-based feature as one of most important feature types. Our Crysalis results were similar to those from other methods and the final optimal feature sets of MF, PF, and CRYs classes contained a number of AAindex-based features. Additionally, other contributing features included composition of tripeptides and the 20 standard amino acids. Details of the final optimal feature set for each class can be found in the supplementary files at http://nmrcen.xmu.edu.cn/crysalis/Datasets.html.

**Figure 1:**
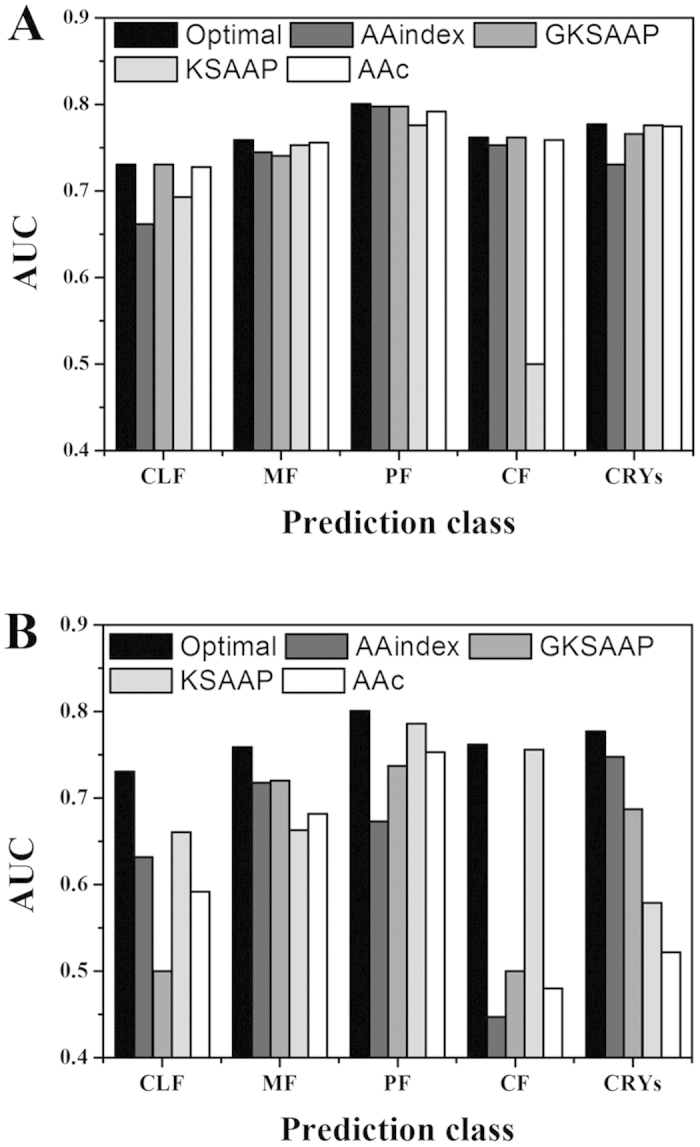
AUC-based performance following removal or inclusion of individual four-feature types in the optimal feature set. The graphs illustrate the impact on prediction performance of the Crysalis first-level models for all five prediction classes. (**A**) Performance of the trained models following removal of the corresponding feature type. (**B**) Performance of the trained models using the individual feature type. All predicted models are compared with the best models that were trained using the optimal feature sets.

We proceeded to evaluate the importance and contribution of the four feature types for each prediction class. First, we examined the influence of iteratively performing each feature type on AUC-based prediction performance by removing this feature type from the final feature set. Furthermore, we analyzed the performance contribution of each feature type on all of the five-class predictions by applying the individual feature types in the optimal feature set. The results are shown in Fig. 1. For CRYs prediction, the important order of the four feature types was AAindex, GKSAAP, KSAAP, and AAc, with AUCs of 0.748, 0.687, 0.579, and 0.522, respectively. For MF prediction, the GKSAAP is the least important feature type contributing to the optimal prediction model. GKSAAP was used to successfully predict PF propensity, resulting in an AUC of 0.737. However, Crysalis did not select any feature-based GKSAAP encoding as a component part of the optimal feature set for the CLF and CF classes. Contrastingly, KSAAP played a decisive role in SVR-model training for those two classes. The importance of KSAAP on the CF class was reflected by the large decrease in AUC scores (from 0.762 to 0.500) when we used the remaining features to train SVR models. It is evident that AAindex-based features are important for the CLF, MF, PF, and CRYs prediction classes.

In summary, this study developed a new feature-encoding method called GKSAAP and found that it played an important role in the prediction of multi-experimental procedures during X-ray crystallography-based structure determination. We hope that this new feature-encoding method can be widely applied to other structural bioinformatics research. A complex subset consisting of four different kinds of feature types was finally selected by multi-step feature selection, which made the prediction models more accurate and robust, except for the CF class. The importance and contribution of the four feature types used in the final feature set can help us understand the process of protein crystallization and enhance protein crystallizability by engineering suitable features in target proteins.

### Performance improvement based on implementation of second-level SVR predictors

Our previous PredPPCrys^3^ demonstrated successful probability outputs for each experimental procedure generated by first-level predictors. The five outcomes CLF, MF, PF, CF, and CRYs form a cascade such that each is correlated with the previous outcomes have been passed or not^3^. For this reason, we also built a second-level SVR predictor (Crysalis II) using five probability outputs from the first-level SVR predictor (Crysalis I) as input features. Crysalis II displayed improvements in performance across all five classes, with improved AUC scores from 0.731 to 0.759, 0.772 to 0.793, 0.796 to 0.801, 0.739 to 0.752, and 0.788 to 0.838 for the classes CLF, MF, PF, CF, and CRYs, respectively. In order to improve SVR-model performance, we optimized all SVR models from both Crysalis I and II using three available kernel types (sigmoid, radial-basis function, and polynomial) with a grid search of two kernel parameters (*C* and γ).

### Performance comparison with other methods

To benchmark the predictive performance of Crysalis, we compared its performance with the three most recently developed tools (PredPPCrys^3^, PPCPred^2^, and XtalPred-RF^17^), as well as other existing methods (ParCrys^7^, OBScore^6^, CRYSTAP2^8^, XtalPred^11^, and SVMCRYs^13^), using previously published independent test datasets^3^. The performance of all predictors was evaluated using AUC, MCC, ACC, SPE, SEN, and PRE measurements and the results are summarized in Table 3. It should be noted that PredPPCrys^3^ and Crysalis were able to predict five-class experimental procedures. Most tools can only predict protein crystallization, but not for all experimental procedures, with the exception of PPCPred^2^, which is capable of four-class prediction. Therefore, we compared Crysalis to the tools capable of providing valid predictions for the procedures of interest. Ultimately, Crysalis achieved AUC scores of 0.759, 0.793, 0.801, 0.752, and 0.838 for the five-class experimental procedures of CLF, MF, PF, CF, and CRYs, respectively.

Most tools focus on predicting the propensity of a sequence to yield CRYs. Therefore, we analyzed and compared this prediction class. The binary classification-based prediction methods for protein crystallizability, including XtalPred, SVMCRYs, XtalPred-RF, and SCMCRYS, were primarily evaluated using MCC scores. They achieved MCC values of 0.244, 0.142, 0.205, and 0.145, respectively, which were lower than those achieved by Crysalis (MCC = 0.435). The real-valued propensity-based prediction method is more robust when performed on the updated datasets than those based on binary classification, given the importance of estimating the difficulty (or successful propensity) of a sequence to yield diffraction-quality crystals. Additionally, some current non-crystallizable proteins may become crystallizable using new protein crystallization technologies in the future^2^,^3^. Our previous tool, PredPPCrys^3^, and Crysalis are real-valued propensity-based predictors and outperform existing tools, including ParCrys^7^, OBscore^6^, CRYSTAP2^8^, and PPCPred^2^.

PPCPred^2^ first introduced four experimental-step predictions and is a SVM-based tool that utilizes comprehensive sets of 36 AAindex-based and sequence-derived predicted structural features (predicted disorder, secondary structure, and solvent accessibility) to estimate protein crystallization propensity^2^. Crysalis outperformed PPCPred^2^ for all four-class experimental procedures, with improvements in prediction performance. Most recently, PredPPCrys^3^ distinguished the process of protein crystallization into five-class procedures by adding the initial class of sequence cloning; these datasets were used in this study. PredPPCrys^3^ adopted a comprehensive set of multifaceted sequence-derived features (amino acid types, compositions, physiochemical properties, predicted structural features, protein features generated from an online web server (PROFEAT)^32^, and multifaceted feature combinations) in combination with a novel multi-step feature-selection strategy to build two-level SVM predictors. Compared to PredPPCrys^3^, Crysalis integrated four sequence-based feature groups, resulting in both high prediction performance and computational efficiency. Results of the independent test show that Crysalis outperformed PredPPCrys^3^ for the classes of CLF and CF, and displayed higher scores for the classes of CLF and CRYs. For PF prediction, Crysalis performance was lower than that of PredPPCrys^3^. However, another advantage of Crysalis is its computational efficiency as compared to three state-of-the-art methods (PPCPred^2^, XtalPred-RF^17^, and PredPPCrys^3^). Its turnaround time for a prediction task (<1s) is significantly shorter than that of all three tools, suggesting a 50-fold speed improvement (PredPPCrys^3^, PPCPred^2^, and XtalPred-RF^17^ required an average of 196, 153, and 84 s, respectively, for completing a protein analysis). Based on these results, Crysalis is more suitable for proteome-wide target selection for protein crystallization and may be applied to develop an analysis tool aimed at computational design by examining the propensity changes associated with target-protein mutations^33^.

### Crysalis web server implementation

**Figure 2:**
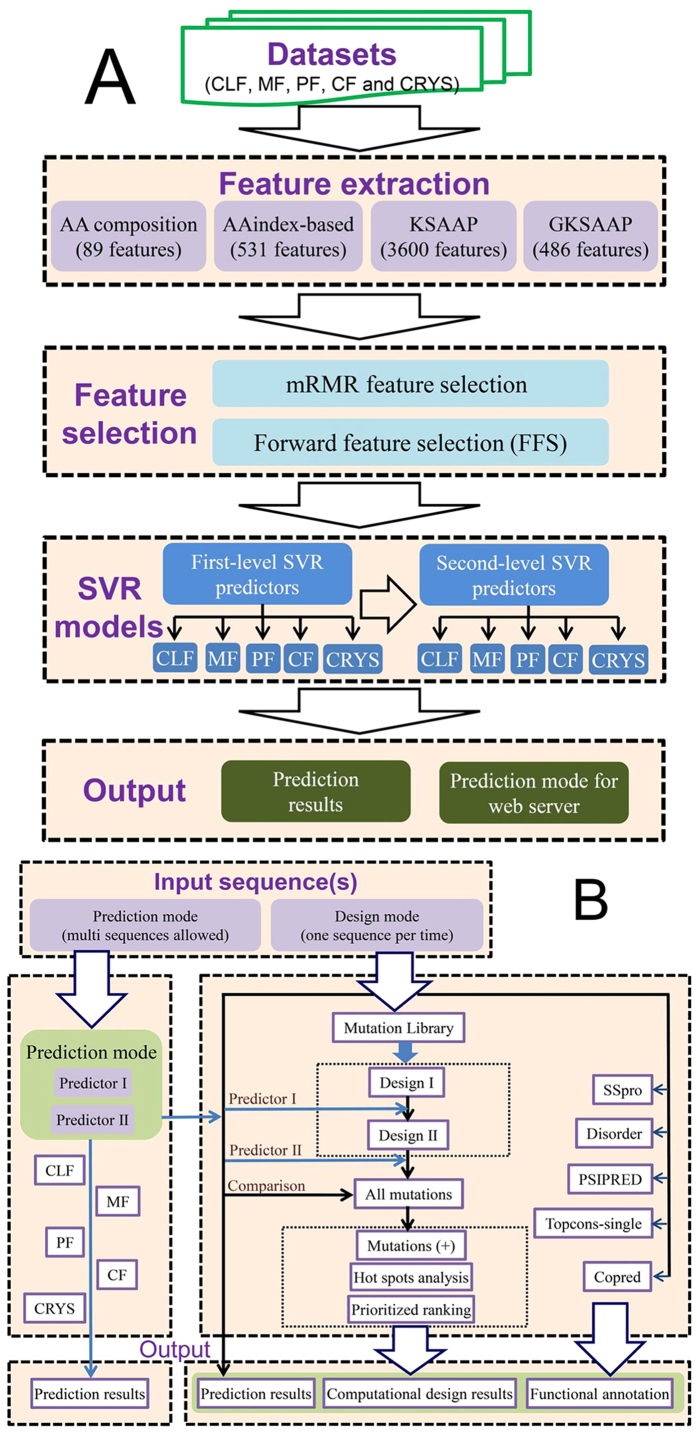
Schematic illustration of the Crysalis prediction mode (**A**) and design mode (**B**).

Crysalis has two built-in modes that support and communicate with each other, i.e. the prediction mode and design mode (Fig. 2B). Two models are used for rapid target selection and computational design, respectively. Examples of its usage and outputs can be found at our website http://nmrcen.xmu.edu.cn/crysalis/FAQ.html. We briefly discuss these two modes in the following sections.

### Prediction mode

The purpose of the prediction mode is to predict the propensity of a target protein to successfully undergo multi-step experimental crystallization procedures based on its amino acid sequence, comprising two-level SVR results (Crysalis I and Crysalis II). It is noteworthy that users can run a batch job (up to 10,000 proteins allowed) to predict the propensity of the target protein to successfully pass any of the five major experimental steps. A typical batch prediction task will require ~10 min before returning prediction results of 5,000 sequences.

### Design mode

The design mode improves the experimental design of those target proteins predicted by Crysalis with a lower propensity score, enabling it to pass a particular experimental procedure for successful crystallization, and ultimately, determination of its structure. Hence, the design mode may be seen as a complementary module to the prediction mode, enabling users to improve crystallization success rates by focusing on the designed mutants with enhanced propensity rather than the wild-type. In design mode, a number of possible single-point mutations are introduced and their corresponding crystallizability scores are estimated by applying regression models from the prediction mode. As a result, Crysalis is able to generate a comprehensive list of designed (or prioritized) mutants for a target protein ranked and annotated with their corresponding propensity scores. The design mode takes Crysalis 5–25 min to comprehensively analyze and return the results of a query sequence, with the computational time primarily depending on amino-acid sequence length. Moreover, the design mode allows users to submit a query for a target protein of interest, returning three resulting outputs:

### Prediction results

The results of protein crystallizability propensities include two-level scores generated by level-1 and level-2 SVR models of the five-class experimental procedures. The results are informative and helpful in estimating protein crystallizability propensity for the wild-type target protein and identifying which experimental procedure(s) might fail in the experimental efforts.

### Computational design results

The results of all recommended protein mutants that are predicted to enhance protein crystallizability propensity following single-point mutation are available for download at the Crysalis website as either residue- or score-based result files. Additionally, this is useful for identifying candidate residues (or sites) whose mutation is potentially helpful for improving protein crystallizability. These results are shown and highlighted as a hotspot map of site non-optimality, where red-colored residues represent candidate sites whose mutation is predicted to be potentially helpful for enhancing protein crystallization propensity. Note that only 20 recommended prioritized mutations will be shown at the output web page, while a comprehensive list of all recommended mutants (in order of increasing predicted propensity scores) can be downloaded as an additional file for follow-up investigations.

### Complementary functional annotations

To facilitate choosing plausible target protein mutants, the Crysalis server integrates and outputs target protein functional annotations, including transmembrane regions, secondary structure, predicted natively-disordered regions, residue solvent accessibility (in terms of exposed or buried status), functional domains (beginning and ending positions), and conserved residues inferred from multiple sequence alignments. Point mutations that occur within a functional domain or those predicted to interrupt functional sites would be disfavored as compared to other mutation options localized outside functional domain regions. These functional annotations are expected to facilitate selection of mutants and hypothesis-driven experimental analysis.

### Complementary functional annotation and analysis of target proteins by Crysalis

Crysalis also provides complementary target protein functional features and annotations (previously detailed), which enable investigation of sequence-structure-function relationships for the purpose of analyzing, designing, and selecting suitable mutations for protein crystallizability. To examine the relationship between structural properties of residues and protein crystallizability, we analyzed site non-optimality (i.e. *C*_i_, see Methods) of a proteome-scale dataset (the independent CRYs test dataset) with respect to secondary-structure types, disorder/order, buried/exposed status, side-chain entropy, charged amino acids, hydrophobicity, and sequence loci (Table 4). Our results revealed that residues in three different secondary-structure types did not display statistical significance relative to site non-optimality; however, residues predicted to be disordered exhibited stronger non-optimality than those predicted to be ordered (*C*_i_ > 0.2 category, 1.64% vs. 0.19%). We also observed that exposed residues have a greater tendency (0.46% vs. 0.23%) to be targets of mutation, consistent with the expectation that protein engineering experiments usually involve exposed residues, owing to disfavored intermolecular interactions caused by exposed amino acids. However, side-chain entropy reduction has proven to be a powerful strategy for protein crystallization design^22^. Here, our results indicate that the majority of residues with higher conformational entropy (KQE) tend to be located at the protein surface and appear to be non-optimal as compared to those buried. Additionally, amino acid charge is another important factor than might influence protein crystallization. We found that residues C, H, N, and R constitute the most ‘designable’ options (Table 5). Specifically, C is often an influencing factor in protein microheterogeneity due to its role in forming incorrect inter- and intra-disulfide bonds. Notably, in terms of the sequence loci, both N-terminal and C-terminal regions (with a length of 20 amino acids) are more amenable to alteration toward the design of protein crystallizability as compared to intermediate regions (0.90% and 2.75%, respectively, vs. 0.14%). This raises the possibility of developing a truncated expression-based experimental strategy for enhancing protein crystallizability.

### Design mode experimental validation

We tested the Crysalis design mode on a proteome-scale protein mutation dataset (*Mut*) recently annotated and extracted from TargetTrack (http://sbkb.org/). The *Mut* dataset includes 11,946 target proteins with 32,126 experimental trials, with each target protein comprised of a wild-type trial and one or more mutations. We applied prediction models to estimate alterations in protein crystallizability, following the incorporation of specific mutations. More specifically, Crysalis predicted the differences in crystallizability propensity between the mutations and the wild-type variant using the Crysalis CRYs prediction model. We only considered a set of targets whose wild-type structure was predicted to be non-crystallizable (score < 0.5). Finally, Crysalis displayed 75.1% accuracy in correctly predicting that the probability change associated with specific mutations was computed as a positive value (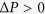). These results suggest that Crysalis could be used in the prediction and computational design of protein crystallization and could be specifically applied to promote engineering target-proteins to enable the crystallization of predicted non-crystallizable proteins.

### Case study

In this section, we provide a case study comprising two application examples to further illustrate the prediction and design utility of the Crysalis webserver.

The target protein enoyl-CoA hydratase/isomerase from *Acinetobacter* sp. ADP1 (NCBI sequence name YP_047189.1 in TargetTrack NYSGXRC-11252j) was non-crystallizable annotated with the status of ‘cloned’ in the database. Crysalis II predicted that the procedures of CLF, MF, CF and CRYs had propensity scores of 0.426, 0.283, 0.471 and 0.484, respectively. To improve protein crystallizability, we further performed a target selection for this protein by applying Crysalis to estimate the protein crystallizability changes of candidate proteins. 100 candidate proteins with a sequence identity of >76% along with an successful experimental example were downloaded from the NCBI Reference Sequence^34^, with the batch prediction results available at http://nmrcen.xmu.edu.cn/crysalis/prediction/Result_344.html. The results suggest that several candidate proteins were predicted to have greater crystallizability than the original YP_047189.1, which can be used for structural determination of the representative 3-D structure of this protein family. In addition, the experimental results provided by TargetTrack show that if the target sequence was replaced by its homologous protein modified from *Acinetobacter baumannii* (NCBI sequence name: WP_004885783) with a sequence identity of 78%, diffraction-quality crystals (PDB entry: 3FDU) would be successfully yielded. Crysalis II predicted that the corresponding propensities of the experimental procedures of this modified protein were 0.551, 0.572, 0.584, and 0.527 for CLF, MF, CF, and CRYs, respectively, which is in a good agreement with the experimental results.

As another example, the nucleoside-diphosphate-sugar pyrophosphorylase from *Vibrio cholerae* RC9 (TargetTrack name: VcR193) was annotated as non-crystallizable with the status of ‘purified’ in the TargetTrack database. Crysalis I accurately predicted that both procedures of PF and CRYs failed with the predicted propensities of 0.236 and 0.468, respectively. Mutagenesis experiments indicate that three single mutations A42R, V122R, and L211E can improve protein crystallizability, reaching 0.238 and 0.473 for the A42R mutant (‘crystalized’), 0.237 and 0.463 for the V122R mutant (‘crystalized’), 0.239 and 0.475 for the L211E mutant (‘diffraction-quality crystal’, PDB entry: 4EVW), respectively. In this case, although the predicted scores of the three mutants were still lower than the cut-off value of 0.5, the crystallizability improvement has led to the successful production of crystals or diffraction-quality crystals. We further applied the Crysalis design mode to analyze the amino acid sequence of this protein. The results show that the propensity changes of CRYs for the A42R, V122R and L211E mutants were 0.005, −0.005 and 0.007, respectively, while their propensity changes of PF were 0.002, 0.001 and 0.003, respectively. The ranking of propensity change CRYs for the L211E mutant was 594/4826, suggesting that a large set of single mutations might be used to further improve its protein crystallizability. The L211E mutation seems to play an important role in stabilizing its 3D structure through the binding of Mg^2^^+^ (Supplementary Fig. S1), which might be a beneficial factor for improved protein crystallization. In summary, the two examples can serve as a useful guide for users to prioritize optimal targets from its homologous proteins using both the prediction and design modes of Crysalis. However, as the first machine learning algorithm-based tool developed for protein crystallizability design, Crysalis still has certain limitations and can be further improved.

## Discussion

A brief review of existing methods for protein crystallization prediction suggests that corresponding machine-learning models exhibit contradictory relationships between prediction performance and computational efficiency. Methods designed to use only sequence-based features achieve reasonable computational efficiency (second scale), but consistently result in poor model performance. However, many recent approaches integrate sequence-derived structure prediction features to train their model, allowing for more accurate prediction performance despite the computational cost (minute scale). Therefore, previous methods appear unsuited for application towards computational design for protein crystallizability, wherein the predictor is required to test propensity changes associated with all probable single-point mutations. Studies indicate that these methods exhibit poor prediction performance and/or computational efficiency. Furthermore, binary classification-based tools, such as XtalPred-RF^17^, cannot report changes in the real-valued propensity that may occur between wild-type and incorporated mutations, making it unsuitable for the computational design of potential mutations.

Here, we developed Crysalis, a novel *in silico* design method for protein crystallizability, based on machine learning. More specifically, Crysalis is able to estimate the propensities of the five major experimental procedures required by X-ray crystallography-based structure determination, which include CLF, MF, PF, CF and CRYs. In particular, the predictors of CLF could be used to predict the propensity for a target protein sequence to be successfully cloned from the original source of DNA. In general, problematic sequences often have high G+C contents, codon bias and/or complex intron/exon structures^35^,^36^. Nevertheless, as a fast and economically efficient technique, well-established gene synthesis has been widely applied, including optimization of codon bias, translation efficiency and oligonucleotide structure for expression in an organism of interest^35^,^37^. As a result, sequence cloning nowadays is often not considered as a major bottleneck for experimental studies. In this regard, if a cloning failure is predicted for a sequence, gene synthesis may be used as an alternative method.

Crysalis utilizes a variety of useful and multifaceted sequence features in combination with a novel GKSAAP-based feature type, further refined to yield a smaller subset of optimal features for each class following multi-step feature selection. This method builds accurate two-level SVR models of five major experimental procedures, facilitating the prediction of real-valued propensities for these procedures. Our method distinguishes itself from previous methods by its increased computational efficiency, as well as its superior or comparable performance to state-of-the-art prediction tools for protein crystallization propensity. Therefore, Crysalis can be used for the selection of crystallizable proteins from a large pool of candidates on a proteome scale. Furthermore, Crysalis SVR models enable the estimation of propensity changes in protein crystallizability associated with all probable single-point mutations, which aids target protein engineering. Previous methods based on the concept of SER^22^,^23^ only discover/resolve one aspect related to protein crystallization failure. Contrastingly, Crysalis designs target-protein mutations according to multifaceted protein features that can be used to resolve different issues that might result in failure to yield diffraction-quality crystals. We validated our design model on a proteome-scale, using proteins currently classified as non-crystallizable, and the results revealed that site non-optimality related to protein crystallization involves biases associated with disorder, solvent-exposed regions, SER, charge, or C-/N-terminal localization. These results are consistent with protein engineering methods employed by experimentalists and the SER concept.

To promote further investigation, the Crysalis design mode integrates several bioinformatics tools to annotate predicted secondary structure elements, residue solvent accessibility, disordered regions, transmembrane regions, functional domains, and conserved sites. Protein crystallization is a complicated process involving several experimental procedures, any of which can result in experimental failure. Crysalis addresses these issues by concentrating on the propensity prediction and computational design of five experimental procedures. Finally, we showed that Crysalis can be applied to the computational engineering of protein crystallizability through proteome-scale validation of experimental mutations recently extracted from TargetTrack. Moreover, a user-friendly web server for target selection (prediction mode) and computational design (design mode) for protein crystallization experiments has been made available. We anticipate that Crysalis will be a powerful tool enabling protein engineering to enhance protein crystallizability, which may accelerate experimental studies and help to determine the crystal structures of biologically important proteins.

## Methods

### Datasets

The benchmark datasets used in this study were created during the course of previous PredPPCrys work^3^ and extracted from the PepcDB website (http://sg.pdb.org)^38^, currently named TargetTrack (http://www.sbkb.org/tt/). The datasets provide five class assignments in terms of experimental crystallization failure or success for the included target proteins, including CLF, MF, PF, CF, and CRYs. Crysalis was developed using the SVR algorithm, its performance evaluated using the benchmark datasets, and further tested using independent test datasets.

In order to evaluate the performance of our design models, we downloaded the most recent experimental datasets from TargetTrack (April 25, 2015), comprising 94,605 protein targets and 944,479 experimental trials. Each target consists of several experimental trials with each trial defined as an objective crystallization trial representing a set of experimental procedures used to crystallize the target^3^. This validation *Mut* dataset was extracted and selected according to the following criteria: (1) The most recent and advanced experimental status was annotated and used for each target. The statuses were grouped into procedures: selected (‘other’, ‘test target’, and ‘selected’), cloned (‘cloned’, ‘biological assay’, ‘biophysical analysis’, and ‘expression tested’), expressed (‘expressed’), soluble (‘soluble’ and ‘membrane protein solubilized’), purified (‘purified’), crystallized (‘crystallized’) and diffraction-quality crystals (‘diffraction-quality crystals’, ‘crystal structure’ and ‘in PDB’). (2) We only collected X-ray crystallography-based experimental trials. (3) To accurately estimate changes in experimental status, we only selected trials with a status of ‘cloned’ or higher. We then removed targets comprising only one experimental trial, because those targets did not contain any mutations in the TargetTrack database. (4) We collected the final status of all trials for each target and selected the target comprising multiple statuses. (5) We randomly assigned the lowest trial as the wild-type variant of the target and removed the trials sharing the same status or protein sequence as the wild-type. (6) Each target contained a trial consisting of the wild-type variant and several mutations (other trials) that promote protein crystallizability. In summary, there are 11,946 target proteins and 32,126 experimental trials (11,946 wild-type proteins and 20,180 mutations) in the *Mut* dataset. This resulted in an average of 1.67 mutations for each target protein. The *Mut* dataset is available at the website http://nmrcen.xmu.edu.cn/crysalis/Datasets.html.

### Feature extraction

Crysalis extracts a comprehensive set of sequence-derived features (4706 features) as candidate features grouped into four main feature types: AAc, AAindex, KSAAP, and GKSAAP. The features used in this study are briefly discussed below:

### AAc-based features include the following 89 features

(i) frequencies of 20 standard amino acids (20 features); frequencies of hydrophobic (F,I,W,L,V,M,Y,C,A), hydrophilic (R,K,N,D,E,P), neutral (T,H,G,S,Q), positively-charged (K,H,R), and negative-charged (D,E), amino acids summarized over the whole sequence (5 features); frequencies of tripeptides with the subgroup of hydrophobic, hydrophilic, and neutral properties (3 × 3 × 3 = 27 features); frequencies of tripeptides with the subgroup of positively-, negatively-, and neutrally-charged properties (3 × 3 × 3 = 27 features); frequencies of 10 residue types based on the chemical groups of their side chains. The 10 chemical groups include phenyl (F,W,Y), carboxyl (D,E), imidazole (H), primary amine (K), guanidine (R), thiol (C), sulfur (M), amido (Q,N), hydroxyl (S,T), and nonpolar (A,G,I,L,V,P) (10 features)^13^.

### AAindex-based features: (531 features)

We used the AAindex1 indices from the AAindex database^24^ to encode the physicochemical properties of 20 standard amino acids and subsequently computed average physicochemical properties over the entire sequence. Finally, we generated a total of 531 features based on the complete indices from AAindex1.

### KSAAP features: (3600 features)

The KSAAP composition developed by Chen *et al.*^27^ was used in this study. For this scheme, the amino acid pairs of 20 standard amino acids include 400 (20 × 20) dimensional features. Moreover, different KSAAPs are also considered, including *k* = 0, 1, 2, 3, and 4 for computing KSAAP features. Additionally, we summarized amino acid pairs whose distance is less than a given *k* (1, 2, 3, or 4). For example, if *k* = 3, then the statistical amino acid pairs would comprise all amino acid pairs of KSAAP_k = 0_, KSAAP_k = 1_, KSAAP_k = 2_, and KSAAP_k = 3_.

### GKSAAP features: (486 features)

For GKSAAP computing, the 20 amino acids were grouped into three classes: low (−1), middle (0), and high (+1), according to the relative physicochemical properties. For example, we can classify the 20 amino acids as negative (low), neutral (middle), or positive (high). More specifically, the following equation is used to classify the 20 amino acids into three groups:


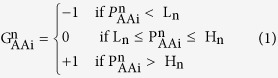


where 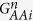 is a grading score of the amino acid *i* (*AA*_i_), for the property *n*, 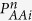 is the numeric value of the property, *n*, of the amino acid *AA*_i_, 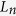 and 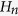 are the low and high cutoffs of the property *n*, respectively. Crysalis takes into account six different types of physiochemical properties, classifying the 20 amino acids into three corresponding subgroups based on their respective types and further encoded the GKSAAP features by varying *k* from 0 to 4. Classification of the 20 amino acids according to the six different types of physiochemical properties is shown in Table 6. Similar to the KSAAP scheme, we also summarized grading amino acid pairs whose distance is less than a given *k*. Finally, 486 features were generated by the GKSAAP encoding scheme, which is proposed for the first time in the present study and encodes nine GKSAAP features for each *k*.

### Crysalis architecture

Crysalis includes a prediction mode and design mode, which support the crystallization propensity prediction and computational design of a target protein, respectively. We describe these two modes in the following two sections.

### Prediction mode

The purpose of the prediction mode is to predict the propensity of a target protein to successfully undergo multi-step experimental procedures based on its amino acid sequence. To address this, we built five classification models to predict the sequence cloning, protein production, purification, and crystallization failure/success propensities, denoted as CLF, MF, PF, CF, and CRYS, respectively^3^. A schematic illustration of the Crysalis prediction mode is presented in Fig. 2A. After extracting a large set of sequence-derived features, we employed a multi-step feature-selection algorithm to select subsets of optimal features for the prediction of each experimental class. In first-step feature selection, we used the mRMR method^39^ to remove redundant and irrelevant features from each feature group of AAindex-, KSAAP- and GKSAAP-based features, and selected the top 100 contributory features for each feature group. In second-step feature selection, we performed the second-step mRMR feature selection on the combining feature sets of the selected 100 AAindex-, KSAAP-, and GKSAAP-based features after first-step feature selection and the remaining set (AAc-based features). In the third-step feature selection, the FFS method^3^,^25^,^26^ was used to select an optimal subset of the best-performing features via 5-fold cross-validation. Finally, 25, 78, 95, 68, and 65 optimal features were selected for training the SVR models of CLF, MF, PF, CF and CRYs, respectively. Similar to PredPPCrys^3^, Crysalis adopts a two-stage strategy to train the five-class prediction models. In particular, the final features selected via feature selection were used as inputs to initially build SVR models of the first-level predictors, termed Crysalis I. Next, prediction outputs by Crysalis I were used as inputs to build second-level SVR models, termed Crysalis II.

### SVR implementation and parameter optimization

We used SVR from the LIBSVM package 2.82^40^ to train and build five-class models for two-level predictors. We used the radial-basis kernel function on the benchmark datasets with default parameters to train SVR models in the process of feature selection and the performance was evaluated via AUC with 5-fold cross-validation. The optimal feature subset for each class was used to build the final model, which was optimized by selecting three available kernels in LIBSVM (sigmoid, radial-basis function, and polynomial) with a grid search of two kernel parameters (*C* and γ). The optimal SVR models for two-level predictors were evaluated on the independent test datasets.

### Design mode

The design mode is available to improve experimental design of those target proteins that are predicted by Crysalis with a lower propensity score to pass a particular experimental procedure toward successful crystallization, and ultimately, determination of its structure. Hence, the design mode may be regarded as a complementary ‘module’ to the prediction mode, improving the crystallization success rate by focusing on the designed mutants having enhanced propensity rather than the wild-type variant.

As shown in Fig. 2B, the computational design of Crysalis was implemented based on the analysis of the predicted protein crystallizability propensity by the prediction mode. First, a library of all possible single-point mutations was generated and their sequence information subsequently used as the input to estimate the corresponding crystallizability propensity of each protein mutant. Crysalis then ranks these mutants based on the estimated changes in protein crystallizability following mutation and identifies mutations that may lead to improved crystallizability. These procedures are described in detail below.

### Estimating protein crystallizability changes upon mutation

The changes in predicted protein crystallizability in response to a given mutation can be computed based on the propensity changes of the predicted experimental procedure (Eq. 2):


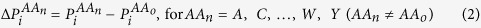


For CRYs prediction, where 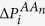 is the crystallizability propensity change upon point mutation of protein residue, *i*, from the wild-type (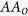) to any of the other 19 amino acids, (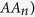, 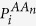 and 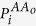 denote the prediction scores of the mutant (*AA*_o_ mutated to *AA*_n_ for the residue *i*) and wild-type sequences using the Crysalis II predictor. Finally, Crysalis is able to generate a comprehensive list of designed (or prioritized) mutants ranked and annotated with their corresponding changes in propensity scores.

### Identifying residues (sites) susceptible to protein crystallizability

In order to identify the key candidate residues amenable to protein engineering capable of improving protein crystallizability, we introduced the score, 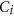, to quantify the degree of non-optimality associated with the amino acid at position *i*, with respect to overall crystallizability:


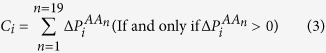


where 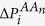 is the corresponding predicted crystallizability change. The 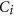 score is the sum of all mutations predicted to increase protein crystallizability at a given position. If mutations are predicted to decrease the propensity of protein crystallizability, 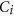 is expected to be close to zero for those residue positions. This situation implies that the wild-type residue should be maintained, as it is regarded as the optimal selection for protein crystallization. However, if the position *i*, results in positive and large values of 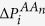, this indicates that the corresponding residue could be an interesting candidate for mutation, due to the associated increase in protein crystallizability.

### Complementary functional annotations of the target protein

Point mutations that occur within a functional domain or interrupt functional sites would be unfavorable compared to other mutation options that localize outside functional domain regions. Such functional annotations are expected to facilitate the selection of mutants and hypothesis-driven experimental analysis. Therefore, Crysalis integrates annotations of transmembrane segments, functional domains, and conserved residues of the target protein generated through the following online web servers: (1) TOPCONS-single^41^ for transmembrane segments, (2) COPRED^42^ for functional domain assignment, (3) PSIPRED^43^ for prediction of the secondary-structure elements of the query protein, including α-helices (H), β-strands (E), and coils (C), (4) natively disordered region in the query protein predicted by DISOPRED 2^44^, and (5) ACCpro 4.1^45^ for solvent accessibility prediction in terms of residue exposure or burial status.

### Performance evaluationj

To evaluate the performance of Crysalis SVR models, we used AUC as the primary measure. In model training and optimization processes, the AUC score is the primary measure for evaluating model performance as suggested by our previous work^3^. In addition to the AUC, we also employed several other measures to evaluate performance of the final Crysalis SVR predictors and compare with other methods:


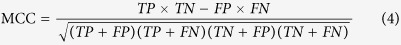



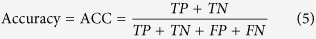



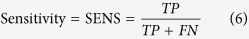



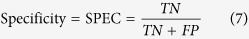



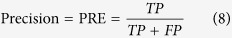


where *TP*, *FP*, *TN*, and *FN* are the numbers of true positives, false positives, true negatives, and false negatives, respectively. Specifically, *TP* and *TN* denote the numbers of correctly predicted successful and failed trials of a given experimental procedure, respectively, while *FP* and *FN* signify the numbers of incorrectly predicted successful and failed trials of a given experimental procedure, respectively.

## Additional Information

**How to cite this article**: Wang, H. *et al.* Crysalis: an integrated server for computational analysis and design of protein crystallization. *Sci. Rep.* **6**, 21383; doi: 10.1038/srep21383 (2016).

## Supplementary Material

Supplementary Information

## Figures and Tables

**Table 1:** Statistics of the final selected features after one-step and two-step feature selection for each class of experimental procedure.

Feature type	CLF		MF		PF		CF		CRYs	
	OFS	TFS	OFS	TFS	OFS	TFS	OFS	TFS	OFS	TFS
AAc	1	0	3	3	1	1	1	1	1	1
Tri-peptide	2	2	6	4	10	9	3	1	9	6
AAindex	7	1	22	20	10	8	1	1	24	21
KSAAP	86	21	19	13	66	64	94	65	26	13
GKSAAP	4	0	50	38	13	13	1	0	40	24
Total	100	24	100	78	100	95	100	68	100	65

Feature selection was performed on the benchmark datasets.

OFS (One-method Feature Selection) denotes results after two-step feature selection (mRMR).

TFS (Two-method Feature Selection) denotes results after three-step feature selection (mRMR+FFS).

AAc: features generated based on 20 standard amino acids.

**Table 2:** Performance comparison of first-level SVR predictors from PredPPCrys and Crysalis.

	Models	Features#	AUC	MCC	ACC(%)	SPE(%)	SEN(%)	PRE(%)
CLF	PredPPCrys	31	0.727	0.339	67.8	62.7	71.4	73.3
	Crysalis	25	0.732	0.334	66.7	68.6	65.5	76.2
MF	PredPPCrys	43	0.777	0.384	70.3	69.6	71.8	50.4
	Crysalis	78	0.767	0.400	70.4	68.4	75.1	49.8
PF	PredPPCrys	54	0.790	0.445	73.8	70.5	75.5	83.3
	Crysalis	95	0.790	0.447	74.4	69.0	77.1	83.4
CF	PredPPCrys	229	0.707	0.289	62.7	74.8	58.8	87.8
	Crysalis	68	0.737	0.329	70.7	73.3	63.1	85.5
CRYs	PredPPCrys	37	0.765	0.309	69.2	69.1	69.3	34.2
	Crysalis	65	0.773	0.326	69.2	68.5	72.2	34.7

Performance of all models were evaluated using the benchmark datasets.

^#^The number of final selected features used for training the first-level SVR predictors.

**Table 3:** Prediction performance comparison of Crysalis and other existing methods.

Experimental step	Method	AUC	MCC	ACC(%)	SPEC(%)	SENS(%)	PRE(%)
CLF	PredPPCrys I	0.711	0.296	65.33	63.58	66.50	73.16
	PredPPCrys II	0.725	0.322	66.54	65.56	67.20	74.44
	Crysalis I	0.731	0.332	66.98	66.60	67.22	75.56
	Crysalis II	**0.759**	**0.365**	**68.34**	**69.95**	**68.34**	**76.85**
MF	PPCPred	0.683	0.334	68.06	67.99	68.22	47.20
	PredPPCrys I	0.772	0.380	69.93	68.21	72.88	49.95
	PredPPCrys II	**0.793**	0.416	71.95	71.36	73.30	52.70
	Crysalis I	0.759	0.377	70.23	69.93	70.99	49.25
	Crysalis II	**0.793**	**0.427**	**73.08**	**73.58**	**73.09**	**54.15**
PF	PPCPred	0.612	0.183	58.83	62.23	57.08	74.57
	PredPPCrys I	0.800	0.460	74.83	70.52	77.02	83.77
	PredPPCrys II	**0.872**	**0.579**	**79.73**	**81.43**	**78.86**	**89.31**
	Crysalis I	0.796	0.436	73.87	67.80	73.87	82.47
	Crysalis II	0.801	0.411	71.22	72.67	70.48	83.48
CF	PPCPred	0.432	−0.014	55.23	32.21	61.24	75.53
	PredPPCrys I	0.712	0.280	67.05	67.65	66.91	89.42
	PredPPCrys II	0.735	0.175	**69.47**	68.89	**69.50**	**97.80**
	Crysalis I	0.739	0.281	65.50	70.59	64.23	89.80
	Crysalis II	**0.752**	**0.337**	62.57	**85.29**	56.93	93.97
CRYs	ParCrys	0.611	0.132	59.66	60.56	55.91	25.40
	OBScore	0.638	0.184	59.28	57.78	65.49	27.14
	CRYSTAP2	0.599	0.123	51.64	48.10	67.78	22.28
	XtalPred	—	0.224	65.04	65.61	62.51	29.31
	SVMCRYs	—	0.142	55.11	52.78	65.70	23.39
	PPCPred	0.704	0.254	63.63	62.09	70.67	29.03
	XtalPred-RF	—	0.205	60.94	59.67	66.41	27.56
	PredPPCrys I	0.770	0.326	69.65	69.30	71.13	35.23
	PredPPCrys II	**0.838**	0.428	76.04	76.21	75.30	42.64
	Crysalis I	0.788	0.339	71.00	70.89	71.41	35.50
	Crysalis II	**0.838**	**0.435**	**76.27**	**76.28**	**76.20**	**42.84**

Performance was evaluated using independent test datasets. Note that most methods (ParCrys, OBScore, CRYSTAP2, XtalPred, and SVMCRYs) only provide one-class prediction (CRYs) and PPCPred includes four-class (MF, PF, CF, and CRYs) predictors. Thus, we only compared the performance of these tools for valid classes. In the case of PredPPCrys, we compared its performance with Crysalis for all five classes.

**Table 4:** Statistical analysis of site non-optimality for protein crystallizability engineering using the independent test dataset for the CRYs class (sequence redundancy removed at 25% sequence identity).

*C*_*i*_ threshold	All^a^(645599)	Secondary structure			Disorder		Buried/Exposed		Side chain entropy^b^		
		Coil(296571)	Helix(260752)	Sheet(88276)	Disorder(66129)	Order(536962)	Exposed(275189)	Buried(370410)	SCE(96423)	SCE_E(74506)	SCE_B(21917)
*C*_*i*_ > 0.005	52.2%	49.8%	56.1%	49.1%	57.3%	52.3%	51.1%	53.2%	36.3%	38.0%	30.4%
*C*_*i*_ > 0.010	32.3%	30.2%	35.4%	29.8%	39.3%	32.2%	31.7%	32.7%	21.4%	22.9%	16.3%
*C*_*i*_ > 0.02	15.3%	14.2%	16.9%	14.6%	22.7%	15.1%	15.9%	15.0%	10.6%	11.6%	7.01%
*C*_*i*_ > 0.05	4.08%	3.92%	4.36%	3.83%	8.80%	3.76%	4.65%	3.67%	3.37%	3.80%	1.90%
*C*_*i*_ > 0.1	1.22%	1.27%	1.23%	1.02%	3.83%	0.99%	1.53%	0.99%	1.26%	1.44%	0.62%
*C*_*i*_ > 0.2	0.33%	0.41%	0.27%	0.22%	1.64%	0.19%	0.46%	0.23%	0.43%	0.48%	0.24%
**Charged Amino acids**			**Hydrophobic**			**Sequence loci**^c^					
**Negative(73537)**	**Positive(83209)**	**Charged(156745)**	**Low(196812)**	**Middle(164236)**	**High(284551)**	**N-terminal (36180)**		**Intermediate(573239)**		**C-terminal(36180)**	
35.4%	55.4%	46.0%	49.6%	51.7%	54.3%	67.5%		50.6%		62.3%	
22.7%	36.0%	29.7%	31.2%	30.3%	34.1%	48.9%		30.4%		45.6%	
12.4%	19.0%	15.9%	15.7%	13.9%	16.0%	29.3%		13.7%		28.1%	
4.45%	6.01%	5.28%	4.42%	3.81%	4.01%	10.8%		3.18%		11.8%	
1.62%	2.35%	2.00%	1.35%	1.30%	1.08%	3.76%		0.78%		5.66%	
0.56%	0.95%	0.77%	0.36%	0.47%	0.23%	0.90%		0.14%		2.75%	

^a^The dataset contains 2,342 proteins comprising of 1,814 proteins currently classified as non-crystallizable. Residue numbers for different groups are shown in brackets.

^b^Statistical analysis of side-chain entropy considered three residues with high conformational entropies (KQE). SCE denotes the number of KQE residues in the entire sequence, while SCE_E and SCE_B denote the numbers of KQE residues annotated to be localized to exposed or buried regions, respectively.

^c^N-terminal and C-terminal denote the initial and final 20 residues located at the N- or C-terminal region of protein sequences. The Intermediate group is comprised of all residues from protein sequences, excluding N-terminal and C-terminal residues.

**Table 5:** Site non-optimality analysis of 20 standard amino acids for protein crystallizability.

AAs	Number	*C*_i_ threshold of site nonoptimality					
		0.005	0.01	0.02	0.05	0.1	0.2
Total	645599	52.2%	32.2%	15.3%	4.08%	1.22%	0.33%
A	57068	53.8%	27.4%	9.68%	1.54%	0.24%	0.21%
C	8137	85.7%	73.8%	45.9%	16.1%	4.48%	0.96%
D	34265	46.9%	29.7%	15.7%	5.05%	1.48%	0.34%
E	39524	25.5%	16.6%	9.55%	3.94%	1.73%	0.75%
F	27004	59.1%	42.3%	24.3%	6.65%	2.00%	0.42%
G	45831	36.9%	18.0%	6.12%	1.23%	0.32%	0.08%
H	14891	50.2%	38.5%	26.2%	12.9%	7.20%	4.02%
I	36964	53.7%	35.5%	17.0%	4.05%	1.02%	0.16%
K	32967	37.0%	20.2%	9.45%	2.77%	1.00%	0.27%
L	68034	59.1%	40.2%	20.3%	5.88%	1.81%	0.51%
M	14613	56.3%	34.7%	15.6%	3.60%	0.82%	0.07%
N	25557	76.7%	53.1%	27.6%	7.09%	1.86%	0.33%
P	29532	45.5%	23.6%	9.33%	1.81%	0.38%	0.04%
Q	24284	53.0%	30.8%	13.8%	3.25%	0.82%	0.11%
R	35632	74.6%	49.5%	24.9%	6.13%	1.55%	0.29%
S	44801	72.5%	45.5%	22.0%	5.34%	1.38%	0.23%
T	35036	44.4%	23.4%	8.66%	1.78%	0.33%	0.03%
V	43817	46.2%	25.9%	10.1%	1.95%	0.49%	0.06%
W	9114	37.0%	23.2%	10.4%	2.40%	0.51%	0.08%
Y	20786	46.0%	25.5%	9.64%	1.76%	0.33%	0.01%

Crysalis performed the computational design of the CRYs class using proteins currently classified as non-crystallizable in the independent test datasets with 25% sequence identity.

**Table 6:** Classification of the 20 amino acids in the GKSAAP encoding scheme according to six different types of physicochemical properties.

Physicochemical property	Low	Middle	High
Accessible surface area	ACGILFV	HMPSTWY	RNDQEK
Side-chain orientation	NDQEK	ARGHPSTWYV	CILMF
Charge	DE	NQCILMFSWTYVAGP	KHR
Hydrogen-bond donors	ADCGST	NQEHILMPV	RKFWY
Hydrophobicity	THGSQ	RKNDEP	FIWLVMYCA
van der Waals potential	ILMFWY	RCQEHKPV	ANDGST
