# Supplementary material for: Crysalis: an integrated server for computational analysis and design of protein crystallization
Source: Sci Rep. 2016 Feb 24;6:21383. doi: 10.1038/srep21383 (PMC4764925; doi:10.1038/srep21383)
Supplement: Supplementary Information [file srep21383-s1.pdf]

Supplementary Data

Crysalis: an integrated server for computational analysis and design of protein crystallization

Huilin Wang[1], Liubin Feng[1], Ziding Zhang[2], Geoffrey I. Webb[3], Donghai Lin[1,*] and Jiangning Song[3,4,5,*]

[1]Department of Chemical Biology, College of Chemistry and Chemical Engineering, Xiamen University, Xiamen 361005, China, [2]State Key Laboratory of Agrobiotechnology, College of Biological Sciences, China Agricultural University, Beijing 100193, China, [3]Faculty of Information Technology, Monash University, Melbourne, VIC 3800, Australia, [4]Department of Biochemistry and Molecular Biology, Faculty of Medicine, Monash University, Melbourne, VIC 3800, Australia and [5]National Engineering Laboratory for Industrial Enzymes, Tianjin Institute of Industrial Biotechnology, Chinese Academy of Sciences, Tianjin 300308, China

1. Supplemental Results

1.1 Computational efficiency

Under the prediction mode, it takes Crysalis a few seconds (<1 s) to complete the job of the propensity prediction of the 5-class experimental procedures, while a typical batch prediction task will require ~10 min before returning the prediction results of 5,000 sequences. Under the design mode, it takes Crysalis 5~25 min to comprehensively analyze and return the results of a query sequence, with the computational time primarily depending on its amino acid sequence length.

1.2 User guide of web server

On the Page of 'Frequently Asked Questions (FAQ)' at our webserver, we provides instructions on how to submit a query sequence to Crysalis, and the meaning of the prediction output, which will facilitate users to use the online web server of Crysalis. The more detailed instructions are available at http://nmrcen.xmu.edu.cn/Crysalis/FAQ.html.

In addition, this web tool is also freely available at Structbioinfor.org Server (http://www.structbioinfor.org/Crysalis/) in Tianjin Institute of Industrial Biotechnology, Chinese Academy of Sciences.

1.3 Case study

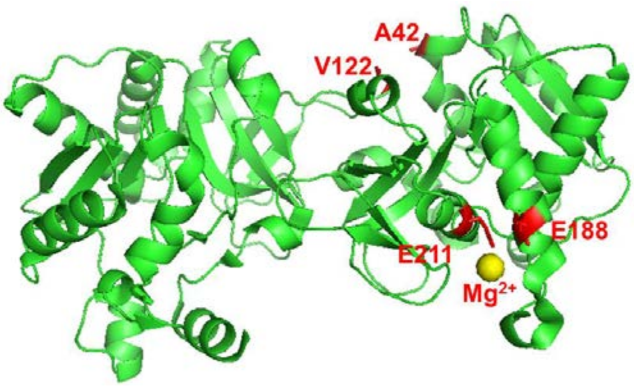

Figure S1. The 3-D structure of nucleoside-diphosphate-sugar pyrophosphorylase (target name, VcR193) from Vibrio cholerae RC9 (PDB entry: 4EVW).
